# ToF-SIMS Analysis of Demineralized Dentin Biomodified with Calcium Phosphate and Collagen Crosslinking: Effect on Marginal Adaptation of Class V Adhesive Restorations

**DOI:** 10.3390/ma14164535

**Published:** 2021-08-12

**Authors:** Francisco Betancourt, Andràs Kiss, Ivo Krejci, Tissiana Bortolotto

**Affiliations:** 1 Division of Cariology and Endodontology, Faculty of Medicine, University Clinic of Dental Medicine, University of Geneva, 1205 Geneva, Switzerland or fmbeta@gmail.com (F.B.); ivo.krejci@unige.ch (I.K.); 2 Department of Quantum Matter Physics (DQMP), Faculty of Sciences, Section of Physics, University of Geneva, 1205 Geneva, Switzerland; andra.kiss@unige.ch

**Keywords:** demineralized dentin, collagen cross-linking, calcium phosphate, riboflavin, universal adhesive, time-of-flight secondary ion mass spectroscopy, ToF-SIMS, marginal adaptation, adhesive interface

## Abstract

This study aimed to assess the effect of biomodification before adhesive procedures on the tooth-restoration interface of class V restorations located in caries-simulated vs. sound dentin, and the quality of dentin surface by time-of-flight secondary ion mass spectrometry (ToF-SIMS). Class V cavities located on cervical dentin were prepared on the buccal surfaces of extracted human molars under the simulation of intratubular fluid flow. Two dentin types, i.e., sound and demineralized by formic-acid, were biomodified with 1% riboflavin and calcium phosphate (CaP) prior to the application of a universal adhesive (Clearfil Universal Bond) in etch and rinse or self-etch mode, and a conventional micro hybrid composite (Clearfil APX). Restorations were subjected to thermo mechanical fatigue test and percentages of continuous margins (% CM) before/after fatigue were compared. Bio modification of dentin surfaces at the molecular level was analyzed by Time-of-Flight Secondary Mass Spectometry (ToF-SIMS). % CM were still significantly higher in tooth-restoration interfaces on sound dentin. Meanwhile, biomodification with riboflavin and CaP had no detrimental effect on adhesion and in carious dentin, it improved the % CM both before and after loading. Etching carious dentin with phosphoric acid provided with the lowest results, leading even to restoration loss. The presence of molecule fragments of riboflavin and CaP were detected by ToF-SIMS, evidencing dentin biomodification. The adhesive interface involving carious dentin could be improved by the use of a collagen crosslinker and CaP prior to adhesive procedures.

## 1. Introduction

Pathologic mineral loss of tooth substance due to chemical (erosion) and bacterial (caries) acid attack are major reasons given by patients to visit the dental practitioner and the two most common reasons for the loss of tooth structure [1]. Moreover, dental caries located in the cervical part of the tooth (root caries) together with dental erosion are by far the two most common oral diseases affecting elder patients [2]. An increased life expectancy, together with the fact that people retain their teeth longer, render the presence of these lesions very common among the elder population. Due to the expected demographic elderly boom in the coming decades, the appearance of lesions in the cervical area will also follow this trend [3].

Carious lesions in the cervical dentin-exposed area appear quite frequently because dental biofilm accumulated in this area causes a critical pH that affects dentin more rapidly than enamel [4]. The situation is aggravated in patients with limited salivary flow, high consumption of acidic beverages, and mouth breathing, as in these cases the time in which acid is in contact with the tooth is increased and remineralization by saliva is hampered [5].

Restorative therapy with resin-bonded restorations still constitutes the first treatment choice and restorations with tight margins are major contributors to clinical success [6,7,8,9,10,11]. Meanwhile, handling of carious dentin beneath restorations is challenging in many clinical situations especially in cases with a limited area for adhesion. Unfortunately, inferior bond strengths were observed in adhesive interfaces involving demineralized carious dentin [12]. This implied that mechanical properties of carious dentin could not be recovered solely by the application of an adhesive system.

Understanding caries as a dynamic process that shifts towards de- or re-mineralization has opened the possibility of new therapies encouraging remineralization of carious tissue and a maximum preservation of dentinal substrate [13]. Acidic carious conditions result in mineral loss and breakdown of collagen network [14]. Therefore, one interesting approach to the treatment of dental caries in the context of minimally invasive dentistry is the reconstitution or repair of carious dentin, which involves two different substrates: organic collagen and inorganic apatite [14,15].

There is a general agreement on the role of type I collagen in dentin’s mechanical properties. This has encouraged many research activities towards the preservation of collagen network by the use of dentin crosslinkers [16]. Glutaraldehyde, genipin, grape seed extract, tannic acid, green tea, and proanthocyanin are both synthetic and natural crosslinking agents that chemically interact with dentin collagen, increasing dentin mechanical properties [17,18,19,20]. Riboflavin (RF: vitamin B_2_) is currently used as a cross-linking agent in ophthalmology to increase mechanical and chemical stability of corneas [21]. It uses photo-oxidation, a physical method, to crosslink collagen [16]. It acts as a photosensitizer that upon photoactivation with UVA or UVB light, reactive oxygen is formed and physical (covalent) cross-links between collagen fibers will be formed through oxidation [22,23]. In addition, antibacterial properties of riboflavin have been demonstrated [24] for inactivation of pathogens like *Staphylococcus aureus*, *Enterococcus faecalis*, *Salmonellas typhi*, *Pseudomonas aeruginosa*, *Escherichia coli*, and *Candida albicans*. From this perspective, the use of RF as a disinfectant could even be beneficial on carious dentin.

Three inorganic apatite-promoter materials known in dentistry for their bio-active properties are glass ionomers, calcium silicates, and calcium phosphates. Glass-ionomers, through their ion exchange with enamel and dentin, are well-known for their ability to support repair and remineralization of dentin that may be left in cavities prior to restoration [25]. Nevertheless, in deep carious lesions approaching the pulp, the acidic environment of glass ionomers, due to the presence of polyacrylic acid, may be aggressive when close to the pulp and too acidic for the ion-rich alkaline environment that is needed for remineralization [25]. Calcium-silicates, the other family of bio-active materials with ion- exchange capability with dentin, result in the formation of calcium hydroxide (CaOH_2_) after setting. Their alkaline caustic effect when in contact with dentin degrade the collagen of interfacial dentin, creating porosities in which mineral ions released from the cement will stimulate cells and promote mineralization [26]. Even if calcium-silicates stimulate pulpal response, they do not precipitate to form hydroxyapatite (HAp).

Calcium phosphates have been incorporated in many biomaterials for dental use such as dentifrices, bleaching agents, restorative composites, adhesive systems, sealants, and orthodontic cements [27], given their ability to hydrolyze quickly to HAp in aqueous media [28]. Regarding aqueous media, although dentinal fluid has a major role in dentin physiology [15] poor attention, until now, has been given by models for caries mineralization proposed in the literature. The use of extracted human teeth with simulation of physiology in living teeth would be an asset, as any liquid-induced mineralization would follow the physiologic path of mineralization, i.e., from pulpal to dentin-enamel junction direction [15].

Time-of-flight secondary ion mass spectrometry (ToF-SIMS) is a powerful analytical tool for studying dentin remineralization as both mineral and organic portions of dentin can be analyzed simultaneously. It is able to characterize chemically complex materials derived from apatite and amino acids (e.g., Ca_2_PO_3_^+^, C_3_H_6_NO^+^, etc.), providing with information about the presence of certain molecules in a specific surface [29]. With this analytical technique the association between mineral change and protein content can be obtained from one measurement [29,30,31,32,33]. The generated ions differ in their flight time; this difference in flight time is measured and their mass is calculated form time of flight data. The mass of the different ions would inform us on their identity based on the chemical composition of the samples’ surface. In the context of dentin remineralization, the presence of molecules (wither entire molecules or molecule fragments) related to apatite-like deposits, collagen, and molecules related to riboflavin can be tracked.

Strengthening demineralized dentin collagen while providing ions favoring HAp deposition might be an interesting minimally invasive alternative to complete caries removal. Therefore, the purpose of this study was to investigate if demineralized dentin can be biomodified through collagen crosslinking and calcium phosphate-doping, prior to adhesive restoration of class V cavities under the simulation of dentinal fluid. The null hypothesis tested was that no differences in terms of marginal adaptation would be observed between the different dentinal treatments.

## 2. Materials and Methods

### 2.1. Specimen Preparation

The materials used in this study consisted of a universal adhesive (CUB: Clearfil Universal Bond Quick, Kuraray Noritake, Japan, batch number 700042, chemical composition: 2-Hydroxyethyl methacrylate (HEMA), bisphenol A-glycidyl methacrylate (Bis-GMA), 10-Methacryloyloxydecyl dihydrogen phosphate (10-MDP), silanated silica, camphorquinone, 1-phenyl-1,2-propanedione (PPD) initiator, ethyl alcohol, water) used in a self-etch or etch and rinse mode, 37% phosphoric acid (H_3_PO_4_: K-Etchant, Kuraray Noritake, Japan), a fine hybrid composite resin (APX: Clearfil APX PLT, Shade A2, Kuraray, Okayama, Japan, batch no. 780062, chemical composition: Bis-GMA, triethylene glycol dimethacrylate (TEGDMA), silanated barium glass filler, silanated silica filler, silanated colloidal silica, dl-camphorquinone, catalysts, accelerators, pigments, others), a solution of 1% riboflavin (RF) [34] prepared by diluting 1 g of riboflavin-5-phosphate (Riboflavin 98%, Alfa Aesar GmbH & Co KG, Karlsruhe, Germany, batch no. H03X008) in 100 mL of distilled water and a calcium phosphate-based material mixed with water in a 1 spoonful/1 drop ratio as recommended by the manufacturer (CaP: Teethmate, Kuraray Noritake Dental Inc., Okayama, Japan, batch no. 000118). For the simulation of intratubular fluid flow, a solution of Phosphate Buffered Saline and horse serum (Bioswisstec AG, Schaffhausen, Switzerland) was used in a ratio of 3:1. The experimental groups (*n* = 8) differed in the way how demineralized dentin was treated prior to the application of the adhesive and composite resin (Table 1). A sample size of 8 was considered sufficient after analyzing, among the 8 groups, the number of samples necessary to detect statistically significant differences between the highest and lowest mean of marginal adaptation before loading, obtaining a power of above 80% (www.ClinCalc.com, post-hoc power calculator).

For this study, extracted human posterior teeth were anonymously collected and thus did not need to be submitted to approval by an ethical committee (Swiss law, Canton of Geneva, Switzerland, Human Research Act, article 2, alinea 2). The selected molars were stored immediately after extraction and refrigerated in 0.1% thymol solution until the beginning of the tests. Twenty-four hours before use they were cleaned with a scaler and pumice, embedded in custom-made specimen holders with their roots in the center using auto-polymerizing resin (Technovit 4071, Heraeus Kulzer, Wehrheim, Germany) and connected to a dentinal fluid simulation device (PAA Laboratories, Linz, Austria). Prior to the holders’ mounting procedure, the root apices were sealed with an adhesive system (Optibond FL, Kerr, Scafati, Italy). To simulate dentinal fluid flow [35,36], a cylindrical hole was drilled into the pulpal chamber approximately at the cement-enamel junction. Then a metal tube with a diameter of 1.4 mm was placed inside the hole until reaching the pulp chamber, and luted with the same adhesive system. Simulation of dentinal fluid flow would be enabled by the use of a flexible hose connecting the sample by its metal tube to a vacuum pump device. The vacuum device contained a solution of phosphate buffered saline and horse serum (3:1 ratio) that would be injected to the pulp chamber and remain inside the dentinal tubules as it is the case with dentinal fluid in vital teeth. Dentinal fluid simulation would be maintained during cavity preparation, adhesive restoration, and thermo-mechanical load.

Saucer-shaped standardized class V cavities were prepared with fine diamond burs (FG 4255/6, Intensiv, Grancia, Switzerland) on the teeth’s buccal cervical area with margins located on dentin. Cavity dimensions (3.5 mm mesio-distal, 3.0 mm occluso-cervical, and 1.5 mm deep) were verified with a periodontal probe. Cavity margins were finished with 25-micron diamond burs and then cavity preparations would be checked under an optical microscope (Wild M5, Wild, Heerbrugg, Switzerland) at 12× magnification to detect marginal imperfections such as fractures or chipping before cavity filling.

In half of the groups (Groups 1 to 4) the class V cavities were prepared in sound (non-demineralized) dentin. In the other groups (Groups 5 to 8) simulation of caries-affected dentin was performed by demineralizing dentin cavities with 10% formic acid for 5 h [37]. Before acid exposure, the crowns were protected by a silicone impression material (President, Light Body, Coltène, Switzerland) placed around the tooth crown with the exception of a window where the cavity was prepared. This enabled demineralization in the area of interest (cavity) while preserving the rest of the crown from acid attack.

Dentin treatment on each group prior to the application of composite resin (APX) is detailed in Table 2.

Dentin was crosslinked by the application of RF for 5 min, that was then air-dried and UV-exposed for 60 s under visible blue light radiation used in the dental field, i.e., 450–470 wavelength [23]. After light-curing of the adhesive system, composite resin (APX) was placed into the cavities in two increments, one cervical and another occlusal, light-cured for 20 s per layer with a light curing device (VALO, Ultradent, Cologne, Germany, power output of 1000 mW/cm^2^_,_ wavelength of 450–470 nanometers verified by a curing radiometer (Demetron, model 100, Danbury, Conneticut, USA) by keeping the light guide at 1 mm from restorations’ surface. After light curing restorations were polished with flexible discs of different grain size (SofLex Pop-On, 3M ESPE, Seefeld, Germany) until visualizing the line corresponding to restoration margins. These margins would be then quantified by Fe-SEM analysis in terms of the absence of gaps or percentages of continuous margins (% CM).

### 2.2. Thermo Mechanical Loading

Samples were then stored in the dark at 37 degrees for one week before starting the thermo mechanical fatigue test. They were subjected to simultaneous thermal and mechanical loading (TML) consisting of 600,000 loading cycles and 1500 thermal cycles at 5 and 50 °C in a custom-made chewing simulator consisting of eight hermetic loading chambers connected to a cold and warm water bath [7,38]. Mechanical load cycles were transferred to the center of the samples’ occlusal surface with a frequency of 1.7 Hz and a maximal load of 49 N by using a natural lingual cusp taken from an extracted human molar and mounted on a metallic support that enabled its placement in the chewing simulators’ chamber.

### 2.3. Quantitative Margin Analysis

Immediately after completion of the polishing procedure (before loading) and after the thermo mechanical fatigue test (after loading), the teeth were cleaned with prophylaxis brush and toothpaste (Signal anti-caries, Unilever, Switzerland). Then, impressions of each restoration were taken with a polyvinylsiloxane material (President light body, Coltène/Whaledent AG, Altstätten, Switzerland). Impressions were then poured with epoxy resin (Epofix resin, Struers, Ballerup, Denmark) to obtain resin replicas that would be gold-coated prior to margin analysis. The computer-assisted quantitative analysis of restoration margins was performed in a field emission scanning electron microscope (Fe-SEM, Sigma 300 VP, Carl Zeiss Microscopy GmbH, Jena, Germany) at 200× magnification by using a custom-made module programmed with an image processing software (Scion Image, Scion, Frederik, MD, USA). All specimens were subjected to quantitative margin analysis and examined, in blind, by a trained lab technician. Restoration margins were quantified by the presence of continuous margins, that is, percentages of margins without the presence of gaps or discontinuities.

### 2.4. ToF-SIMS Molecular Characterization

Molecular analysis by TOF-SIMS: Sample preparation consisted of 1 mm dentin slices whose surface was treated with H_3_PO_4_, CaP and RF. Dentin slices were then dehydrated and the top surfaces were observed under a ToF-SIMS mass spectrometer (nanoTOF II, Physical Electronics, Chanhassen, MN, USA). For the measurement a primary ion beam consisting of 30 keV Bi_3_^2+^ ions was used with an unfiltered DC current of 4 nA. The mass spectrum was recorded in positive ion mode in the mass range between m/z 5–1850. The imaged area was 200 × 200 µm and 30 frames were collected resulting in an ion dose of 2.76 × 10^11^ ions/cm^2^. For analyzing the TOF-SIMS data the TOF-DR version 3.2.0.5 software (Physical Electronics, Chanhassen, MN, USA) was used. The mass spectra were mass calibrated using common small organic fragments (C_2_H_3_^+^, m/z 27.0235; C_3_H_5_^+^, m/z 41.0391; C_4_H_7_^+^, m/z 55.0547).

### 2.5. Statistical Analysis

Statistical analysis for data of marginal adaptation was performed with a specific software (SPSS for Macintosh, version 26). Non-parametric Kruskal–Wallis and post-hoc test with Bonferroni correction was run to detect differences in percentages of continuous margins (% CM) between the eight groups both before and after loading. The confidence level was set to 95%.

## 3. Results

All restorations survived the fatigue test with the exception of two samples in Group 7 and one sample in Group 8 that detached from the cavity; the particularity of these two groups (7 and 8) was that carious dentin was etched with H_3_PO_4_ prior to the application of riboflavin, CaP, the universal adhesive and composite resin.

**Figure 1:**
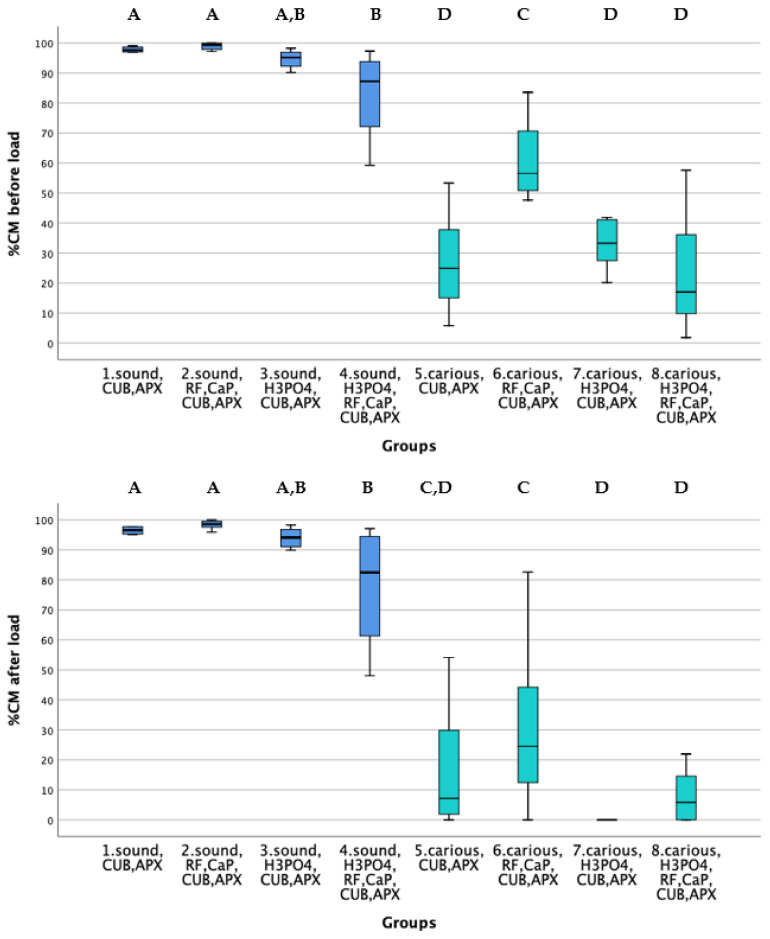
Box-plots resulted from Kruskal-Wallis analysis illustrating the percentages of continuous margins before (upper graph) and after thermo mechanical loading (lower graph). Groups 1 to 4 correspond to treatments on sound dentin and Groups 5 to 8 to treatments on carious dentin. Groups connected by different A, B, C and D letters (post-hoc test with Bonferroni correction) are significantly different at the *p* = 0.05 level. Abbreviations: H_3_PO_4_: 37% phosphoric acid, RF: Riboflavin, CaP: calcium phosphate-containing material, CUB: Clearfil Universal Bond Quick universal adhesive, APX: Clearfil APX composite resin.

Significant differences were observed between the eight groups both before/after loading (Kruskal–Wallis, *p* = 0.000), box-plots are presented in Figure 1. The effect of dentin type was significant and the groups with % CM closest to 100, that is, 100% of gap-free margins, were the ones in which restorations were located in sound dentin (Groups 1 to 4). No significant differences were observed between Groups 1 and 2 (post-hoc test, before loading *p* = 0.319, after loading *p* = 0.296) meaning that the addition of riboflavin and calcium phosphate before adhesive procedures had no adverse effect on marginal adaptation (Gr 2).

Box-plots from Group 5 to 8 represent restorative procedures in caries-simulated dentin. Both before and after loading (Figure 1) the highest % CM were observed in Group 6; this was the group in which carious dentin was doped with RF and CaP before adhesive restorative procedures. In view of the low results observed after loading, the sole application of an adhesive system on carious dentin (Group 5) could not improve marginal adaptation. The worst and statistically similar performing groups were the ones in which carious dentin was etched with H_3_PO_4_ prior to the different dentinal treatments, as shown in Figure 1 (Groups 7 and 8, before loading *p* = 0.546 and after loading *p* = 0.425), with the observation of %CM below 20% after loading and even for some samples, the occurrence of restoration loss.

**Figure 2:**
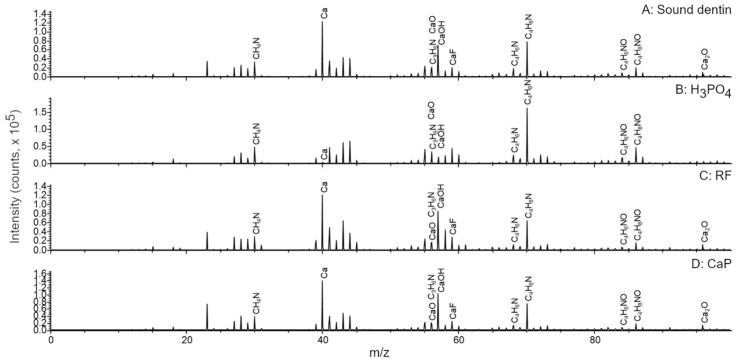
**A to D.** Comparison of the ToF-SIMS mass spectra of dentin after different treatments between m/z 0 and 100.

**Figure 3:**
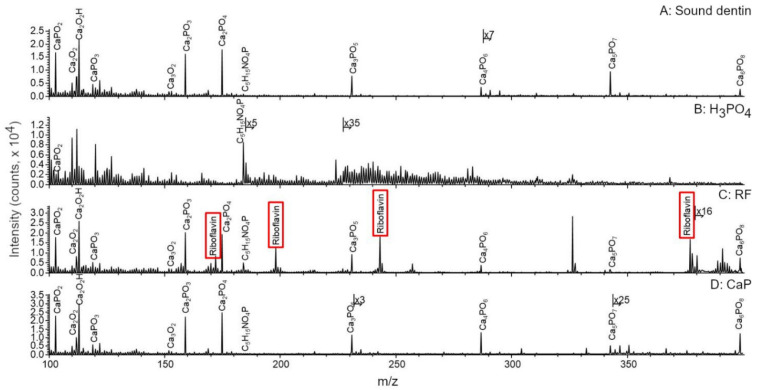
**A to D.** Comparison of the ToF-SIMS mass spectra of dentin after different treatments between m/z 100 and 400. C: Spectra corresponding to the presence of riboflavin (RF) is marked in red rectangle.

Dentin surfaces were analyzed by ToF-SIMS (Figure 2 and Figure 3). Sound dentin (Figure 2A and Figure 3A), riboflavin treated sample (Figure 2C and Figure 3C) and the CaP treated sample (Figure 2D and Figure 3D) contained apatite, which was evidenced by the presence of a series of apatite related ions such as the Ca^+^ ion or the Ca_x_O_y_^+^ and Ca_x_PO_y_^+^ ion series identified in the mass spectra of these samples.

These ions were almost completely missing from the H_3_PO_4_ treated sample (Figure 2B and Figure 3B). The main ions detected were mostly organic fragments after the acid treatment, such as the phosphatidylcholine headgroup at m/z 184.0851 that usually signals the presence of cells, because phosphatidylcholines are the main constituents of cell membranes. Another examples are several small fragments such as ions at m/z 30.0354 (CH_4_N^+^), 56.0510 (C_3_H_6_N^+^), 68.0504 (C_4_H_6_N^+^), 70.0679 (C_4_H_8_N^+^), 84.0463 (C_4_H_6_NO^+^), and 86.0651 (C_4_H_8_NO^+^) generally attributed to the presence of amino acids [39]. The primary source of the latter ions is most likely the type I collagen in the samples as it is the most abundant structural protein in dentin [29]. Finally, the mass spectrum of the riboflavin treated sample (Figure 3C) contained the protonated molecular ion of riboflavin at m/z 377.1362 (C_17_H_21_N_4_O_6_^+^) and several riboflavin related fragments at m/z 243.0829 (C_12_H_21_N_4_O_2_^+^), m/z 198.0606 (C_11_H_8_N_3_O^+^), and m/z 172.0841 (C_10_H_10_N_3_^+^), as expected. After the different dentinal treatments, molecular fragments of the previously mentioned molecules were detected on the surface, evidencing that biomodification occurred on dentin.

**Figure 4:**
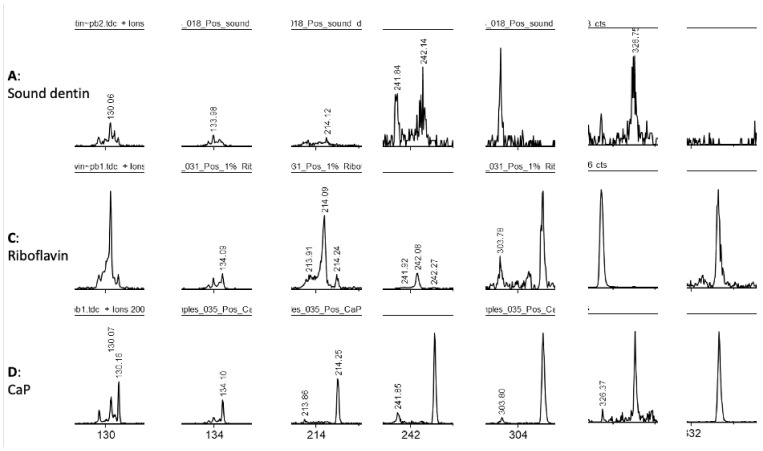
**A, C and D.** Comparison of the ToF-SIMS mass spectra of dentin after different treatments. Specific peaks after the different treatments with riboflavin and CaP are absent from sound dentin, indicating that biomodification took place on dentin surface.

Additionally, a few ions were exclusively detected from the riboflavin or the CaP treated samples but not from the surface of the sound dentin (Figure 4). These ions might be indicating either physical adsorption onto dentin surface, either the occurrence of biological changes in the samples.

**Figure 5:**
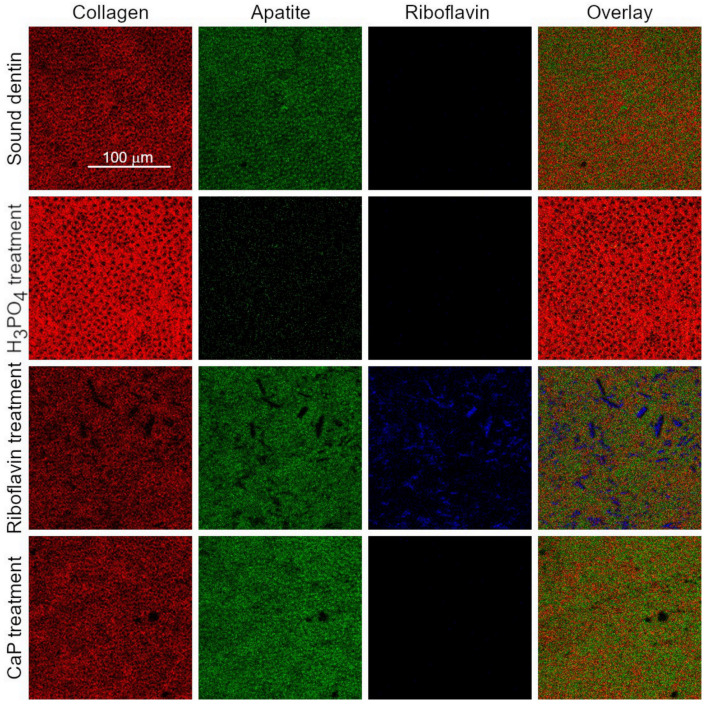
Distributions of fragment ions of collagen (red), HAp-derived Ca (green), riboflavin (blue) and overlay (red-green-blue) at the four different detin surfaces as recorded by ToF-SIMS.

Since ToF-SIMS is a molecular imaging technique, we were able to additionally plot the distribution of the previously mentioned components in the four samples. For the visualization of apatite distribution, the sum of Ca^+^, the Ca_x_O_y_^+^ and Ca_x_PO_y_^+^ containing ions were plotted, collagen was visualized by summing the amino acid related fragments listed in the previous paragraph while for riboflavin the sum of the molecular ion and the fragment at m/z 243.0829 was used. The comparison of the distribution of these main constituents between the four samples revealed a few interesting facts (Figure 5). The riboflavin treated sample contains both collagen and apatite. Additionally, riboflavin also stays on the sample surface but riboflavin does not have a homogenous distribution on the surface; instead it is forming discrete crystals. The images of the other three samples show the lack of riboflavin, as expected. The lack of signal at the selected two ions proves that these ions are good markers for the riboflavin distribution as these mass channels only contain minimal interference in the non-riboflavin treated samples. The H_3_PO_4_ treated sample almost exclusively contains collagen, most of the apatite related ions are completely gone as already was seen from the mass spectrum. The lack of apatite in the sample means that the collagen structures are clearly visible compared to the other samples where the collagen structures are somewhat obscured by the apatite. The apatite distribution is rather homogenous in the sound dentin, the CaP and the riboflavin treated samples and it seems to have the maximum signal where the collagen has the lowest signal. Additionally, both the apatite and collagen signals are suppressed where the riboflavin crystals are located, probably because ToF-SIMS only samples the top 1–2 nm of a sample surface.

## 4. Discussion

The paradigm shift in restorative dentistry towards a minimally invasive approach has completely changed the manner in which carious dentin is handled, especially when its complete removal could compromise pulp vitality [40,41]. Caries-affected dentin is a remanent substrate that is often needed to deal with, especially in cases of penetrant or circumferential cervical caries, in which complete caries removal is not possible without substantially weakening the tooth. In such cases the preservation of carious tissue might be necessary to avoid more invasive restorations or even tooth extraction. Therefore, the purpose of this study was to assess if simulated-carious dentin could be biomechanically improved before restorative procedures and if marginal adaptation of class V restorations could be improved. Based on the results after thermo mechanical fatigue test, the null hypothesis had to be rejected, as significant differences were detected between the different groups.

Class V cavity margins were located on dentin to mimic the clinical location of root caries lesions. Carious dentin was simulated by demineralizing the specimens with formic acid, in agreement with what has been done in previous studies [42]. Half of the groups were performed in sound dentin as this is the ideal dentin situation in terms of marginal seal [43]. The other half were located in carious dentin that would be bio modified, to assess its biomechanical recovery when challenged against a thermo mechanical fatigue test.

Regarding collagen crosslinking, riboflavin (RF) was selected over other crosslinkers [44] as it has shown to significantly decrease the activity of matrix metalloproteinases, abundantly present in caries-affected dentin and responsible of the degradation of adhesive interfaces over time [45,46]. RF is a well-known water-soluble vitamin that is available in daily foods, due to its biocompatibility it has been extensively used for the treatment of ophthalmologic diseases [21], even an antibacterial effect of RF has been observed after its application in corneal collagen [47] and beneficial effects have been reported on dentin [16]. In this study exposed collagen due to demineralization was crosslinked by applying a riboflavin-containing solution, in order to “stabilize” the collagen network prior to mineral nucleation. Recent findings found that the combined use of RF and CaP could improve mechanical properties of demineralized dentin [23]. Other studies have also found beneficial effects of RF on dentin collagen matrix such as an improvement of mechanical stability, mechanical properties and biodegradation resistance [34,48]. In the context of caries, a recent study observed the ability of RF to prevent mineral loss by enabling additional collagen crosslinking to dentin collagen [49].

A CaP powder made of pure tetracalcium phosphate and dicalcium phosphate anhydrous was used in this study as this is one, if not the only, calcium phosphate powder clinically available in its purest form and with remineralizing properties on dentin [50]. When mixed with water, the solution precipitates into HAp as the final product [51]. The decision to add riboflavin to the dentin protocol was based on the positive findings previously reported in terms of dentin collagen reinforcement due to crosslinking [23,52]. Ahn and colleagues [53] also reported an increase of bond strength when applied on carious-affected dentin prior to an adhesive. Therefore, the hypothesis tested in this study was that if carious-dentin is affected by mineral loss and collagen degradation, its mechanical behavior might be improved when both situations (mineral loss and collagen degradation) are reverted.

Etching carious dentin with H_3_PO_4_ prior to adhesive restoration resulted in the lowest results of marginal adaptation and even the loss of some restorations in Groups 7 and 8. An explanation to these observations could be that the additional removal of minerals from dentin substrate due to acid etch besides the minerals that were already removed by formic acid when simulating carious lesions, might have resulted in an almost complete mineral-depleted collagen layer. It is possible that the layer of denatured collagen on top of the dentin surface could have impeded the diffusion of CaP into dentin, blocking any beneficial mineral gain into its structure [26]. The fact that dentin demineralization exceeded adhesive penetration could favor collagenolytic activity, contributing to the degradation of the adhesive interface [54].

In this study, marginal adaptation of restorations located on biomodified carious dentin was an indirect indicator of mechanical recovery, without assessing up to which depth changes in the mineral profile took place across the dentin surface. In this sense, a recent study found a significant increase in microhardness 150 microns deeper from carious dentinal surfaces treated with a crosslinking agent and a calcium phosphate-based compound [55]. Another study found a depth of mineral deposition of 30 microns [56].

Among the groups with restorations on carious dentin (Gr 5 to 8), marginal adaptation in Group 6 was the highest both before and after loading (Figure 1). A previous study compared sound vs. carious dentin by synchrotron radiation and found that at the initial stages of dentin demineralization collagen remains unaffected, providing nucleation sites that are useful for remineralization [57]. In the context of our study, it is possible that dentin collagen in demineralized dentin could benefit from the cross-linking effect of riboflavin, favoring the stabilization of newly formed apatite crystals. The fact that marginal adaptation was improved through a more stress-resistant adhesive interface was, in our understanding, due to an increased collagen stiffness due to crosslinking and not just to the presence of RF on the surface. Similar to previous work [26], in this study no biomimetic analogs of non-collagenous matrix proteins (NCP) were used to enable dentin repair. The dentin organic matrix is constituted by about 10% of NCP [29]. The fact that precipitation of calcium phosphate onto residual crystals induces lesion repair, instead of nucleation of minerals on the organic matrix of dentin [56], could serve to explain our better results in the groups in which dentin was doped with CaP prior to adhesive procedures. This might explain the higher percentages of marginal adaptation in the group in which carious dentin was biomodified before adhesive procedures (Group 6), in respect to the group in which carious dentin was only treated with an adhesive system (Group 5).

ToF-SIMS proved to be an advanced high-resolution surface characterization technique for interfacial characterization in adhesive dentistry. The major advantage of the technique is that it is able to identify and visualize the distribution of molecular fragments on dentin at the same time and, in turn, it enables better understanding of these systems by linking their behavior and changes in their chemical composition. In demineralized dentin there was a consistent absence of Ca (Figure 2, Figure 3 and Figure 4). This may explain the low percentages of marginal adaptation observed in the groups where carious dentin was etched with the universal adhesive in its etch and rinse mode (prior H_3_PO_4_ etch). Mineral-depleted dentin was probably incompletely infiltrated by the adhesive system, in addition to the poor effect of 10-MDP monomer on dentin HAp, which was missing due to the effect of the acid. This is of clinical relevance, as it shows that when dealing with carious dentin, further etching might be detrimental for the quality of the adhesive layer.

## 5. Conclusions

Under the experimental conditions tested in this study, in class V restorations located on sound dentin, the highest percentages of marginal adaptation, close to 100%, were observed when the universal adhesive was used in its self-etching mode (Groups 1 and 2). Dentin biomodification with RF crosslinker and CaP had no adverse effect on marginal adaptation. In restorations located on caries-simulated dentin, the sole application of the adhesive system (Group 5) delivered significantly lower results in respect to the group in which dentin was biomodified with RF crosslinker and CaP (Group 6). Etching caries-simulated dentin with H_3_PO_4_ resulted in almost the entire degradation of restoration margins after loading (Groups 7 and 8), even leading to restoration detachment from the cavities.

## Figures and Tables

**Table 1:** Restorative procedures undertaken in class V cavities of each group are marked with an x. Groups in sound (1 to 4) dentin and groups in caries-simulated dentin (5 to 8).

	Sound Dentin	Caries-Simulated Dentin	H_3_PO_4_ etch	RF + CaP	CUB	APX
Gr 1	x				x	x
Gr 2	x			x	x	x
Gr 3	x		x		x	x
Gr 4	x		x	x	x	x
Gr 5		x			x	x
Gr 6		x		x	x	x
Gr 7		x	x		x	x
Gr 8		x	x	x	x	x

Abbreviations: H_3_PO_4_: 37% phosphoric acid, RF: 1% Riboflavin, CaP: calcium phosphate-containing material, CUB: Clearfil Universal Bond Quick (universal adhesive), APX: Clearfil APX (composite resin).

**Table 2:** Description of restorative procedures in class V cavities of each group.

**Gr 1: CUB + APX in sound dentin**
CUB was applied on dentin surface, air-dried and light-cured for 10 s with a LED Curing Light (VALO, Ultradent, Cologne, Germany, power output: 1000 mW/cm^2^, wavelength: 450–470 nm) at a distance of 1 mm from the light source output.
**Gr 2: RF + CaP + CUB + APX in sound dentin**
Dentin was brushed with RF for 5 min, gently air-dried and UV-exposed with the same light-curing unit as mentioned above for 60 s. Then the CaP-containing material was applied for 2 min, rinsed and air-dried. CUB was applied on dentin surface, air-dried and light-cured for 10 s.
**Gr 3: H3PO4 + CUB + APX in sound dentin**
Dentin was etched with H_3_PO_4_ for 10 s, rinsed and air-dried. CUB was applied on dentin surface, air-dried and light-cured for 10 s.
**Gr 4: H3PO4 + RF + CaP + CUB + APX in sound dentin**
Dentin was etched with H_3_PO_4_ for 10 s, rinsed and air-dried. Dentin was then brushed with RF for 5 min, gently air-dried and UV-exposed for 60 s. Then the CaP-containing material was applied for 2 min, rinsed and air-dried. CUB was applied on dentin surface, air-dried and light-cured for 10 s.
**Gr 5: CUB + APX in caries-simulated dentin**
CUB was applied on dentin surface, air-dried and light-cured for 10 s.
**Gr 6: RF** **+ CaP + CUB + APX in caries-simulated dentin**
Dentin was brushed with RF for 5 min, gently air-dried and UV-exposed for 60 s. Then the CaP-containing material was applied for 2 min, rinsed and air-dried. CUB was applied on dentin surface, air-dried and light-cured for 10 s.
**Gr 7: H3PO4 + CUB + APX in caries-simulated dentin**
Dentin was etched with H3PO4 for 10 s, rinsed and air-dried. CUB was applied on dentin surface, air-dried and light-cured for 10 s.
**Gr 8: H3PO4 + RF + CaP + CUB + APX in caries-simulated dentin**
Dentin was etched with H_3_PO4 for 10 s, rinsed and air-dried. Dentin was then brushed with RF for 5 min, gently air-dried and UV-exposed for 60 s. Then the CaP-containing material was applied for 2 min, rinsed and air-dried. CUB was applied on dentin surface, air-dried and light-cured for 10 s.

Abbreviations: H_3_PO_4_: 37% phosphoric acid, RF: Riboflavin, CaP: calcium phosphate-containing material, CUB: Clearfil Universal Bond Quick universal adhesive, APX: Clearfil APX composite resin.

## Data Availability

The data presented in this study are available under request to the corresponding author TB.
